# A Synthetic Pseudo-Rh: NO_x_ Reduction Activity and Electronic Structure of Pd–Ru Solid-solution Alloy Nanoparticles

**DOI:** 10.1038/srep28265

**Published:** 2016-06-24

**Authors:** Katsutoshi Sato, Hiroyuki Tomonaga, Tomokazu Yamamoto, Syo Matsumura, Nor Diana Binti Zulkifli, Takayoshi Ishimoto, Michihisa Koyama, Kohei Kusada, Hirokazu Kobayashi, Hiroshi Kitagawa, Katsutoshi Nagaoka

**Affiliations:** 1 Elements Strategy Initiative for Catalysts and Batteries, Kyoto University, 1-30 Goryo-Ohara, Nishikyo-ku, Kyoto 615-8245, Japan; 2 Department of Applied Chemistry, Faculty of Engineering, Oita University, 700 Dannoharu, Oita 870–1192, Japan; 3 Department of Applied Quantum Physics and Nuclear Engineering, Kyushu University, Motooka 744, Nishi-ku, Fukuoka 819-0395, Japan; 4 INAMORI Frontier Research Center, Kyushu University, Motooka 744, Nishi-ku, Fukuoka, 819-0395, Japan; 5 Department of Hydrogen Energy Systems, Kyushu University, Motooka 744, Nishi-ku, Fukuoka 819-0395, Japan; 6 Division of Chemistry, Graduate School of Science, Kyoto University, Kitashirakawa-Oiwakecho, Sakyo-ku, Kyoto 606-8502, Japan

## Abstract

Rh is one of the most important noble metals for industrial applications. A major fraction of Rh is used as a catalyst for emission control in automotive catalytic converters because of its unparalleled activity toward NO_x_ reduction. However, Rh is a rare and extremely expensive element; thus, the development of Rh alternative composed of abundant elements is desirable. Pd and Ru are located at the right and left of Rh in the periodic table, respectively, nevertheless this combination of elements is immiscible in the bulk state. Here, we report a Pd–Ru solid-solution-alloy nanoparticle (Pd_x_Ru_1-x_ NP) catalyst exhibiting better NO_x_ reduction activity than Rh. Theoretical calculations show that the electronic structure of Pd_0.5_Ru_0.5_ is similar to that of Rh, indicating that Pd_0.5_Ru_0.5_ can be regarded as a pseudo-Rh. Pd_0.5_Ru_0.5_ exhibits better activity than natural Rh, which implies promising applications not only for exhaust-gas cleaning but also for various chemical reactions.

Rh is an important element that exhibits excellent activity in numerous catalytic reactions^1^,^2^,^3^,^4^,^5^,^6^,^7^. However, the abundance of Rh is only one-fifth of Pt, and it is one of the least abundant elements in the Earth’s crust^8^,^9^. Such rarity makes the price of Rh very volatile and high; for example, Rh reached a record price of $210,000 per kilogram in 2008^10^. The high cost of Rh limits its applicability. The vast majority of Rh (more than 80% of the Rh production in the world^9^) is used for emission-control catalysts in vehicles with modern internal combustion engines; these catalysts have contributed to reducing smog and acid rain in developed countries. Such catalysts, referred to as three-way catalysts, simultaneously eliminate NO_x_, CO, and residual hydrocarbons, which are the major pollutants in exhaust emissions. Rh plays an important role in NO_x_ reduction as a component of the catalytic converter^1^,^2^,^11^,^12^. Because of the drastic growth in automobile demand in developing countries and increasingly stringent regulations of exhaust emissions from vehicles, the development of an inexpensive and highly active NO_x_ reduction catalyst has become increasingly important. Thus far, extensive effort has been devoted to decreasing the Rh content in NO_x_ reduction catalysts^11^,^12^,^13^,^14^,^15^,^16^,^17^; however, to the best of our knowledge, no research on the development of alternative Rh-free catalysts has been reported in the literature.

Pd and Ru are the immediate right and left neighbour of Rh in the periodic table, respectively, and are much less expensive than Rh. On the basis of our concept “interelement fusion”^18^ where solid-solution alloying offers new properties by changing the compositions and/or combinations of alloys’ constituent elements, we expect that a homogeneous mixture of Pd and Ru, i.e. Pd–Ru solid-solution alloy nanoparticles (NPs), will result in a less expensive alternative with characteristics similar to those of Rh. However, this approach faces a challenge, because binary combinations of Pd-Ru are well known to be immiscible at the atomic level and form segregated domain structures in the bulk at all temperatures up to their melting points^19^,^20^,^21^. Recently, Kusada *et al*. have successfully synthesized Pd-Ru NPs over the entire compositional range via a chemical reduction method based on “non-equilibrium synthesis” and “nano-size effect”^22^. In the work, solid solution alloying of Pd and Ru enhanced several properties of NPs. However, it is not uncovered whether Pd-Ru NPs show comparable catalytic performance to that of Rh in the reaction which is invaluably catalysed by Rh. Therefore, the application of the Pd-Ru NPs for NO_x_ reduction is interesting. Here, we report the Pd_0.5_Ru_0.5_ NPs catalyst exhibiting excellent NO_x_ reduction activity, which is a most crucial properties of three-way catalysts^1^,^2^,^11^,^12^, with its electronic structure similar to that of Rh. In other words, Pd_0.5_Ru_0.5_ NPs behave like synthetic pseudo-Rh. This strategy is in line with direction of “elements strategy” driven in the world^23^.

## Results

### Preparation of Pd_*x*_Ru_1-*x*_ NPs and supported catalysts

**Figure 1:**
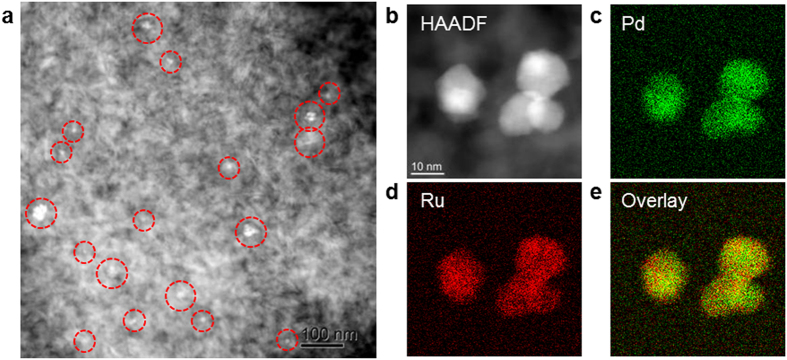
γ-Al_2_O_3_-supported Pd_0.5_Ru_0.5_ solid-solution nanoparticles. (**a,b**) HAADF–STEM images of γ-Al_2_O_3_-supported Pd_0.5_Ru_0.5_. (**c–e**) EDX mapping images of Pd (green) (**c**) and Ru (red) (**d**), and the superposed Pd and Ru element maps (**e**).

Pd_*x*_Ru_1-*x*_ NPs were synthesized via a previously reported chemical reduction method using Pd and Ru precursors. Monometallic Pd, Ru, and Rh NPs were also prepared using the same procedure. The Pd_*x*_Ru_1-*x*_, as well as NPs of Pd, Ru, and Rh (equivalent to 1 wt% of the catalyst), were deposited onto γ-Al_2_O_3_ supports (Fig. 1a) via a standard impregnation method^22^. High-angle annular dark-field scanning transmission electron microscopy (HAADF–STEM) image and elemental maps using energy-dispersive X-ray spectroscopy (EDX) of the γ-Al_2_O_3_-supported Pd_0.5_Ru_0.5_ NPs are shown in Fig. 1. As shown in Fig. 1a, a few primary NPs are forming small secondary particles and those secondary NPs were well dispersed over γ-Al_2_O_3_ support. Figure 1b shows a magnified view of loaded NPs. One can find the overlap of NPs that appear as brighter region in the figure. In addition, the EDX maps of Pd, Ru, and their superpositions (Fig. 1c–e) reveal that Pd and Ru atoms were homogeneously dispersed throughout the NPs. Crystalline structure of Pd_0.5_Ru_0.5_ NPs are confirmed to be fcc-rich, but contains hcp phase also. As the Pd ratio in Pd_x_Ru_1-x_ increases, fcc phase becomes more dominant. Charge transfer from Pd to Ru at the surface of Pd_x_Ru_1-x_ alloy was also demonstrated by X-ray photoelectron spectroscopic measurement. Further details are reported elsewhere^22^.

### Catalytic performance for NO_x_ reduction

**Figure 2:**
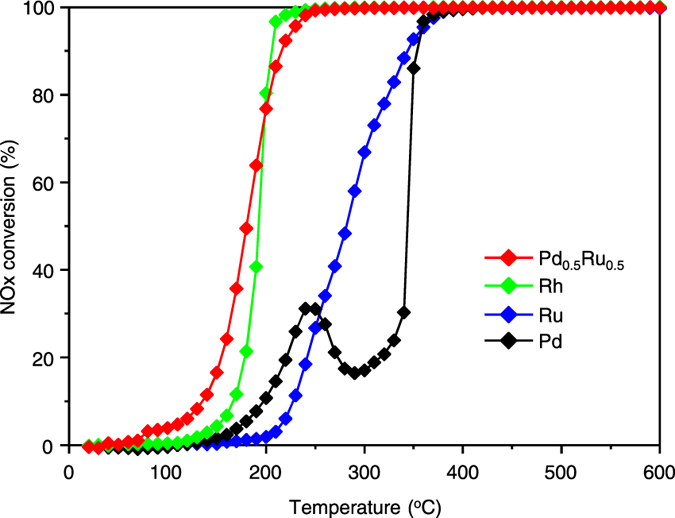
NO_x_ reduction activity for Pd_0.5_Ru_0.5_ solid-solution nanoparticles and control catalysts. The temperature dependence of NO_x_ conversion for several catalysts.

To investigate the catalytic activity of the Pd_0.5_Ru_0.5_ NPs for NO_x_ reduction, we carried out activity tests using a fixed-bed flow reactor. Pelletized γ-Al_2_O_3_-supported Pd_0.5_Ru_0.5_ NPs were packed into a tubular reactor, and we supplied a reaction mixture simulating automotive exhaust with a theoretical air-to-fuel ratio such that the CO and the C_3_H_6_ in the fuel were stoichiometrically combusted by NO and O_2_. The catalyst bed was heated from ambient temperature to 600 °C at a constant rate of 10 °C min^−1^, and the effluent gas was analysed during the heating. For comparison, γ-Al_2_O_3_-supported Pd, Ru, and Rh NPs were also tested under the same conditions (Fig. 2). The NO_x_ conversion rate for Pd_0.5_Ru_0.5_, Ru, and Rh NPs increased smoothly with increasing temperature. By contrast, the Pd NPs exhibited a small peak in their conversion rate at 250 °C, followed by an increase in activity starting from approximately 300 °C, as has been frequently reported in previous studies^24^,^25^. An inspection of Fig. 2 reveals that the Pd_0.5_Ru_0.5_ NPs exhibited equal or greater NO_x_ reduction activity when compared with Rh NPs, which have been considered the most active NO_x_ reduction catalyst. At low temperatures, in particular, the Pd_0.5_Ru_0.5_ NPs exhibited excellent NO_x_ reduction activity that exceeded that of the Rh NPs. The temperatures corresponding to 50% NO_x_ conversion (T_50_) for the Pd_0.5_Ru_0.5_, Rh, Ru, and Pd NPs were 180, 193, 282, and 344 °C, respectively, demonstrating that the Pd_0.5_Ru_0.5_ NPs exhibited the highest NO_x_ reduction activity among the investigated catalysts. Influence of reaction temperature on concentrations of gasses containing nitrogen atom is displayed in Supplementary Fig. S1. N_2_ was major product and NOx was almost completely converted to N_2_ above 350 °C. In addition, N_2_O was also produced from 200 °C. Selectivity of N_2_O increased with increase in the temperature by 250 °C but decreased gradually above the temperature. The catalytic activity of the physical mixture of Pd and Ru (0.5 wt% of Ru NPs and 0.5 wt% of Pd NPs) was also measured for comparison (Supplementary Fig. S2); these mixed NPs exhibited slightly better NO_x_ reduction activity than the unmixed, single-component Pd and Ru NPs. However, the temperature dependence of the catalytic activity for the physical mixture of NPs differed from that of the Pd_0.5_Ru_0.5_ NPs and the activity of the mixture was substantially lower than that of the Pd_0.5_Ru_0.5_ NPs. These results demonstrate that the atomic-level alloying of Pd and Ru in the NPs plays a key role in the emergence of their excellent NO_x_ reduction activity.

### Electronic structure of Pd_0.5_Ru_0.5_ NPs

**Figure 3:**
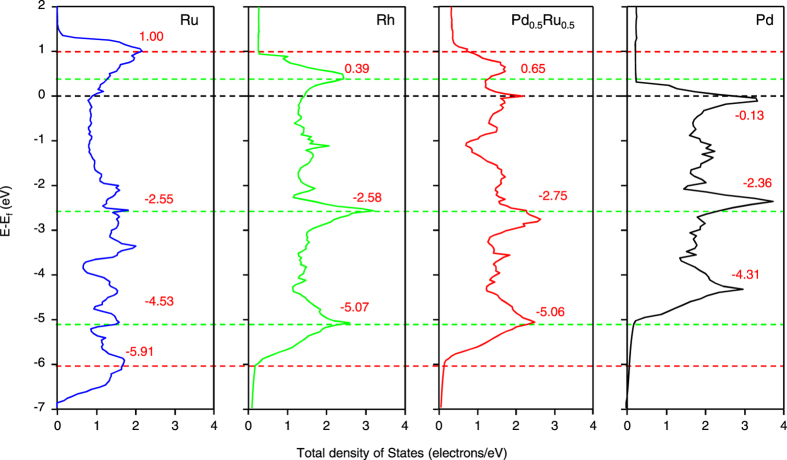
Density of states of Rh, Pd_0.5_Ru_0.5_, Ru, and Pd. Dashed lines are drawn for comparison: Black dashed line is for Fermi energy, green for three major peaks in density of states of Rh, and red for top and bottom of *d*-like band of Rh.

The dissociative chemisorption of NO is well known to be the first step in NO reduction and is also known to be favoured on the Rh surface^2^,^11^. Both the electron donation from the NO 5σ orbital to the Rh 4*d* orbital and the electron back-donation from the Rh 4*d* orbital to the anti-bonding NO 2π orbital, which lead to weakening of the NO bond, have been reported to be important for the activation of the NO molecule for dissociation^26^,^27^,^28^. Therefore, we used first-principles methods to investigate the electronic structure, i.e., the density of states (DOS), of the Pd_0.5_Ru_0.5_ alloy system and the Rh, Pd, and Ru NP systems (Fig. 3). The Rh energy band ranges from approximately +1 eV to −6 eV, with a maximum peak intensity of approximately 3 electrons/eV and three characteristic peaks at +0.39, −2.58, and −5.07 eV. These features are clearly observed in the Pd_0.5_Ru_0.5_ band structure, which differs substantially from those of the Pd and Ru systems. Estimation of *d*-band center in relative to Fermi energy was −2.866 and −2.821 eV for Pd_0.5_Ru_0.5_ and Rh, respectively, while those for Pd and Ru are estimated as −2.306 and −3.075 eV, respectively. Inspection of the partial DOS of Pd and Ru in the Pd_0.5_Ru_0.5_ NPs readily shows that the alloying does not result in a simple linear combination of the electronic structures of Pd and Ru but rather gives rise to an Rh-like electronic structure (Supplementary Fig. S3). This result indicates that the Pd_0.5_Ru_0.5_ alloy can be regarded as a pseudo-Rh in terms of its bulk electronic structure, which should lead to a high catalytic activity similar to that of Rh. Furthermore, the similarity between the Rh and Pd_0.5_Ru_0.5_ DOS observed here suggests that the DOS of a target monometal can be reproduced via the alloying of other elements. To see the correlation between Rh-like electronic structure and the NO_x_ reduction activity, NO adsorption properties onto fcc Pd_0.5_Ru_0.5_(111) as well as Rh(111) are investigated as a first step to screen the activity toward NO molecule. Supplementary Fig. S4 shows the relation between the NO adsorption energy and the N-O bond distance. Compared to the N-O distance of gas phase NO molecule, i.e. 1.172 Å, elongations of N-O distance is observed for all the cases investigated, indicating the activation of NO molecule for the decomposition. Four points for NO adsorption on Rh(111) surface correspond to ontop, bridge, fcc-3-fold, and hcp-3-fold sites, respectively. One can see a general tendency that activation is more evident for bridge than ontop, 3-fold than bridge site adsorption, corresponding to the stronger backdonation in such order. NO adsorption properties on Pd_0.5_Ru_0.5_ shows a complex feature. NO adsorption on Pd ontop site is weaker in adsorption energy and Ru ontop is stronger, while the adsorption on both ontop sites are associated with only a slight activation of NO molecule in terms of N-O distance. For other cases of bridge and hollow-site adsorption, we can see a general trend like Rh, i.e. stronger adsorption energy is associated with the higher activation of NO molecule. Further, it is noted that we can find greater activations of NO molecule on Pd_0.5_Ru_0.5_ at a certain configuration investigated.

### Influence of Pd–Ru composition on catalyst performance

**Figure 4:**
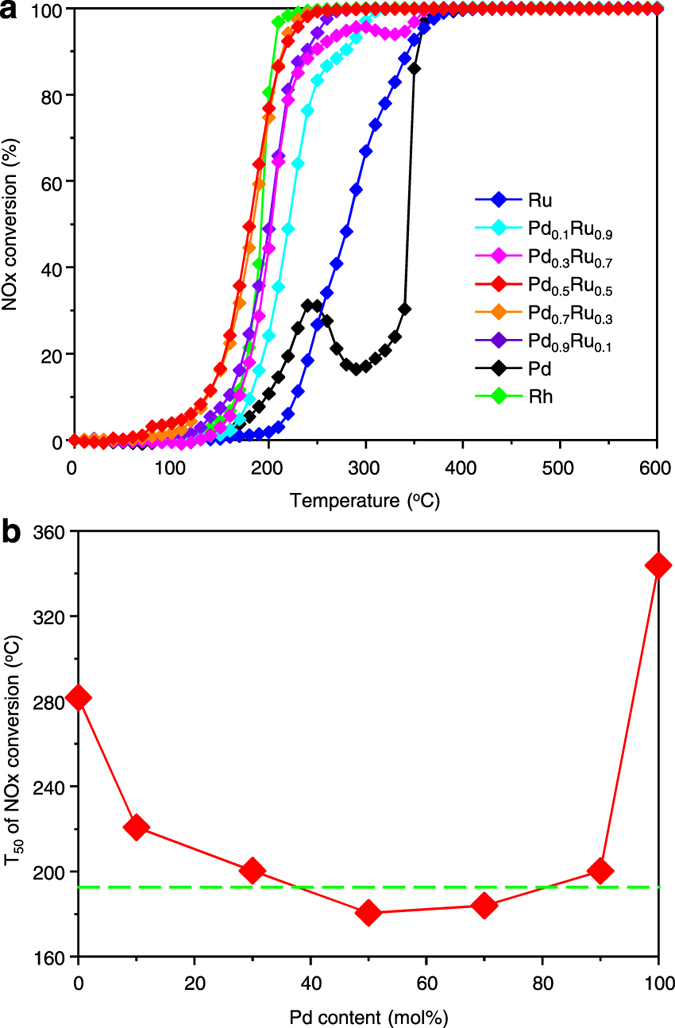
Influence of the atomic ratio of Pd_x_Ru_1-x_ on NO_x_ reduction activity. (**a**) The temperature dependence of NO_x_ conversion for Pd_*x*_Ru_1-*x*_. (**b**) Temperatures corresponding to 50% conversion of NO_x_ (T_50_) in Pd_*x*_Ru_1-*x*_. The dashed line (green) is the T_50_ of Rh.

To investigate the influence of the Pd_*x*_Ru_1-*x*_ composition on the NPs’ activity towards NO_x_ reduction, we carried out activity tests using γ-Al_2_O_3_-supported Pd_*x*_Ru_1-*x*_ NPs with various Pd–Ru compositions (*x* = 0, 0.1, 0.3, 0.5, 0.7, 0.9, 1) (Fig. 4a). Note that mean diameters of the alloy NPs investigated are in the similar range, i.e. ca. 10 nm, except for the Ru as shown in Supplementary Table S1. The T_50_ for NO_x_ conversion observed for all of the Pd_*x*_Ru_1-*x*_ NPs was lower than those of the monometallic Pd and Ru NPs (Fig. 4b). Thus, these results reveal a positive alloying effect over the whole solid-solution composition range. Furthermore, the NO_x_ reduction activity of the Pd_*x*_Ru_1-*x*_ NPs changes continuously with the atomic ratio of Pd and Ru, showing an inverse volcano-type behaviour. In particular, Pd_*x*_Ru_1-*x*_ with *x* = 0.5 and 0.7 exhibited excellent NO_x_ reduction activities that exceeded that of Rh. DOS of Pd_*x*_Ru_1-*x*_ in Supplementary Fig. S5 shows that features of DOS of Rh, i.e. band width, positions and intensities of three characteristic peaks, are retained in the composition calculated. As reported in the preceding work^22^, the fcc phase becomes richer as the Pd composition in Pd_*x*_Ru_1-*x*_ increases. In addition, the DOS of hcp phase of Pd_0.5_Ru_0.5_ is different from that of Rh as shown in Supplementary Fig. S3. Thus, the high activity of Pd_*x*_Ru_1-*x*_ with *x* = 0.5 and 0.7 can be interpreted to originate from the similarity of DOS of Pd_*x*_Ru_1-*x*_ in fcc phase, while we admit the potential complex factors behind the observed activity. It is well known that the commodity prices of Pd and Ru are substantially lower than that of Rh^9^, thus Pd_*x*_Ru_1-*x*_ can contribute to the cost reduction of NO_x_ reduction catalyst system. Considering that the price of Ru is lower than Pd, Pd_0.5_Ru_0.5_ NPs represent a promising new NO_x_ reduction catalyst that costs one-third as much as Rh. A drastic activity enhancement in the case of the Pd_*x*_Ru_1-*x*_ NPs was also observed for the elimination of CO and hydrocarbon (C_3_H_6_), which are other major pollutants in exhaust emissions (Supplementary Figs S6,S7).

## Conclusion

In summary, we developed Pd_*x*_Ru_1-*x*_ NPs with excellent NO_x_ reduction activity compared to that of Rh, which is currently the most active and widely used NO_x_ reduction catalyst. DOS features similar to those of the Rh DOS were observed for bulk Pd_0.5_Ru_0.5_, indicating that the catalytic activity of Pd_0.5_Ru_0.5_ towards NO_x_ reduction is a consequence of its Rh-like electronic structure. These findings predict that Pd_*x*_Ru_1-*x*_ NPs have potential applications as catalysts for various reactions currently catalysed by Rh. The alloying of immiscible elements provides a new direction for the design of novel materials for simultaneous enhancement of catalytic activity and reduction of the materials costs. Our concept of designing materials with desirable and suitable properties via the atomic-level mixing of elements was demonstrated for Pd_*x*_Ru_1-*x*_ NPs, and this approach should be applicable to numerous other combinations of elements and various classes of catalytic reactions, thereby initiating a new scientific field of study.

## Methods

### Synthesis of NPs and supported catalysts

For a typical synthesis of Pd_*x*_Ru_1-*x*_ NPs (*x* = 0.5), poly(*N*-vinyl-2-pyrrolidone) (PVP, 444 mg, MW ≈ 40 000, Wako) was dissolved in triethylene glycol (TEG, 100 mL, Wako) and the resulting solution was heated to 200 °C under magnetic stirring. Meanwhile, K_2_[PdCl_4_] (163.4 mg, Aldrich) and RuCl_3_·*n*H_2_O (131.1 mg, Wako) were dissolved in deionized water (40 mL). The resulting aqueous solution was then slowly added to the TEG solution, which was maintained at 200 °C during the addition for 20 to 40 min. After cooling to room temperature, the prepared NPs were separated by centrifugation. Other Pd_*x*_Ru_1-*x*_ (*x* = 0.1, 0.3, 0.7, 0.9) NPs were prepared by controlling the molar ratio between Pd^2+^ and Ru^3+^ions.

Ru, Rh, Pd, and Pd_*x*_Ru_1-*x*_ alloy NPs and a physical mixture (Ru and Pd NPs) supported on γ-Al_2_O_3_ catalysts were prepared by wet impregnation. Each NP sample (equivalent to 1 wt% of γ-Al_2_O_3_) was ultrasonically dispersed in purified water. A high-purity γ-Al_2_O_3_ support (AKP-GO15, Sumitomo Chemical) that had been precalcined at 800 °C for 5 h was placed into each aqueous NP solution, and the suspended solutions were stirred for 12 h. After being stirred, the suspended solutions were heated to 60 °C and then dried under vacuum. The resulting powders were maintained at 120 °C for 8 h to completely remove water.

### High-resolution TEM (HRTEM), HAADF–STEM, and EDX analyses

Samples were dispersed in ethanol, dropped onto a carbon-coated copper grid, and dried by exposure to ambient conditions for 24 h. HRTEM, HAADF–STEM, and EDX mapping images were collected using a JEM-ARM200F (JEOL) transmission electron microscope operated at 200 kV.

### Theoretical calculations

All calculations were performed using the VASP^29^,^30^,^31^,^32^. The crystal structures of bulk Rh (fcc), Pd (fcc), Ru (hcp), and Pd_0.5_Ru_0.5_ (fcc/hcp) were reproduced using four-atom unit cells with periodic boundary conditions for both fcc and hcp lattices. We prepared three-layers 2 × 2 supercell slab models to study the surface properties. 20 different configurations are prepared for Pd_0.5_Ru_0.5_ surface model to see the influence of configurations of Pd and Ru on the adsorption properties. For each configuration, all possible adsorption sites are tested as the initial structure. The geometries for bulk Rh, Pd, Ru, and the Pd_x_Ru_1-x_ alloys were optimized under the generalized gradient approximation (GGA) with the Perdew-Burke-Ernzerhof (PBE) exchange-correlation functional^33^. Cut off energy is set to be 400 eV and the projector-augmented wave method^34^,^35^ is adopted. Monkhorst–Pack *k*-point grid sampling^36^ was used with the *k*-points of 14 × 14 × 14 and 7 × 7 × 1 for optimization of bulk and surface models, respectively. The DOS were analysed with further refined condition with the cut off of 700 eV and *k*-points of 23 × 23 × 23.

### Catalytic activity tests

Powders of the γ-Al_2_O_3_-supported catalysts were pressed into pellets at 52 MPa for 5 min. They were then crushed and sieved to obtain grains with diameters of 250–500 μm. For each test, 200 mg of catalyst grains was loaded into a tubular quartz reactor (internal diameter = 7 mm), and quartz wool was then packed around the catalyst. In addition, a k-type thermocouple was inserted into the catalyst bed to measure the temperature for feedback control of the system. A feed-gas mixture containing NO (1161 ppm), CO (5750 ppm), C_3_H_6_, (467 ppm), O_2_ (5050 ppm), H_2_ (1760 ppm), CO_2_ (12.5%), and He (balance) was passed over each catalyst (total flow rate = 200 mL min^−1^; space velocity = 60 000 h^−1^). The gas mixture was designed to mimic the air-to-fuel ratio of automotive emissions. The catalyst bed was heated from ambient temperature to 600 °C at 10 °C min^−1^ under the flowing reaction mixture. The outlet gas composition was continuously monitored with a chemiluminescence NO_x_ analyser (VA3000, HORIBA and Model 42*i*-HL, Thermo Fisher Scientific), a non-dispersive infrared gas analyser (VA3000, HORIBA), and gas chromatograph (490 Micro GC, Agilent Technologies).

## Additional Information

**How to cite this article**: Sato, K. *et al*. A Synthetic Pseudo-Rh: NO_x_ Reduction Activity and Electronic Structure of Pd–Ru Solid-solution Alloy Nanoparticles. *Sci. Rep*. **6**, 28265; doi: 10.1038/srep28265 (2016).

## Supplementary Material

Supplementary Information
