# Supplementary material for: A Synthetic Pseudo-Rh: NOx Reduction Activity and Electronic Structure of Pd–Ru Solid-solution Alloy Nanoparticles
Source: Sci Rep. 2016 Jun 24;6:28265. doi: 10.1038/srep28265 (PMC4919684; doi:10.1038/srep28265)
Supplement: Supplementary Information [file srep28265-s1.pdf]

Supplementary Information

A Synthetic Pseudo-Rh: NO_x Reduction Activity and Electronic Structure of Pd–Ru Solid-solution Alloy Nanoparticles

Katsutoshi Sato[1,2], Hiroyuki Tomonaga[2], Tomokazu Yamamoto[3], Syo Matsumura[3,4], Nor Diana Binti Zulkifli[5], Takayoshi Ishimoto[4], Michihisa Koyama[4,5]*, Kohei Kusada[6], Hirokazu Kobayashi[6], Hiroshi Kitagawa[4,6]*, and Katsutoshi Nagaoka[2]*

[1] *Elements Strategy Initiative for Catalysts and Batteries, Kyoto University, 1-30 Goryo-Ohara, Nishikyo-ku, Kyoto 615-8245, Japan*

[2] *Department of Applied Chemistry, Faculty of Engineering, Oita University, 700 Dannoharu, Oita 870–1192, Japan*

[3] *Department of Applied Quantum Physics and Nuclear Engineering, Kyushu University, Motooka 744, Nishi-ku, Fukuoka 819-0395, Japan*

[4] *INAMORI Frontier Research Center, Kyushu University, Motooka 744, Nishi-ku, Fukuoka 819-0395, Japan*

[5] *Department of Hydrogen Energy Systems, Kyushu University, Motooka 744, Nishi-ku, Fukuoka 819-0395, Japan*

[6] *Division of Chemistry, Graduate School of Science, Kyoto University, Kitashirakawa-Oiwakecho, Sakyo-ku, Kyoto 606-8502, Japan*

Catalytic performance for NO_x reduction.

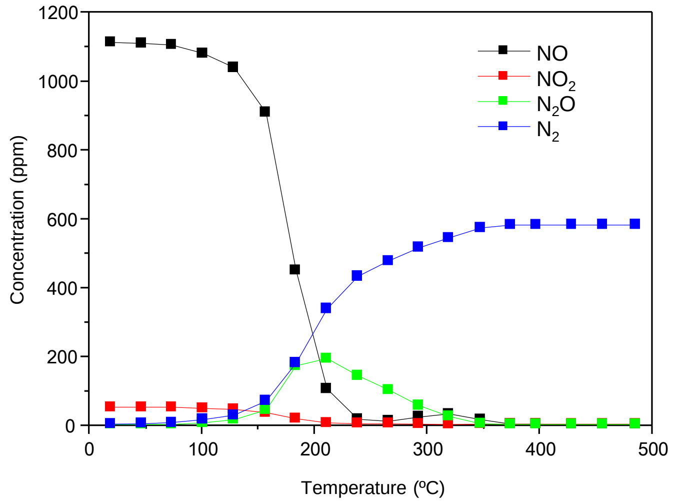

Supplementary Figure S1 | Concentrations of gasses containing nitrogen atom in effluent gas.

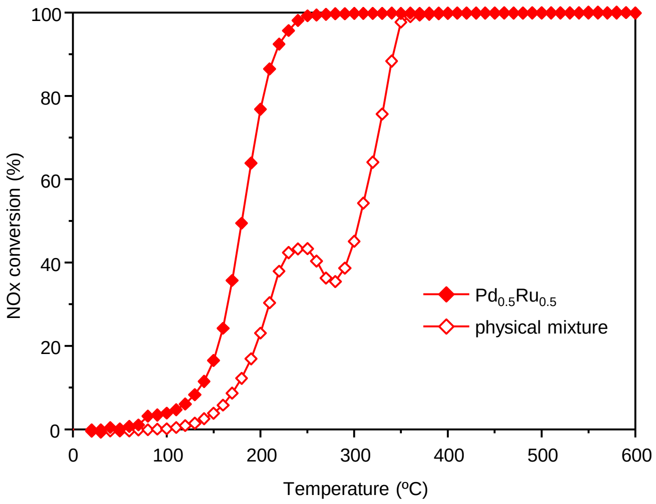

Supplementary Figure S2 | NO_x reduction activity for $Pd_{0.5}Ru_{0.5}$ and a physical mixture of Pd NPs and Ru NPs. Temperature dependence of NO_x conversion for several catalysts.

Electronic structure of $Pd_{0.5}Ru_{0.5}$ NPs.

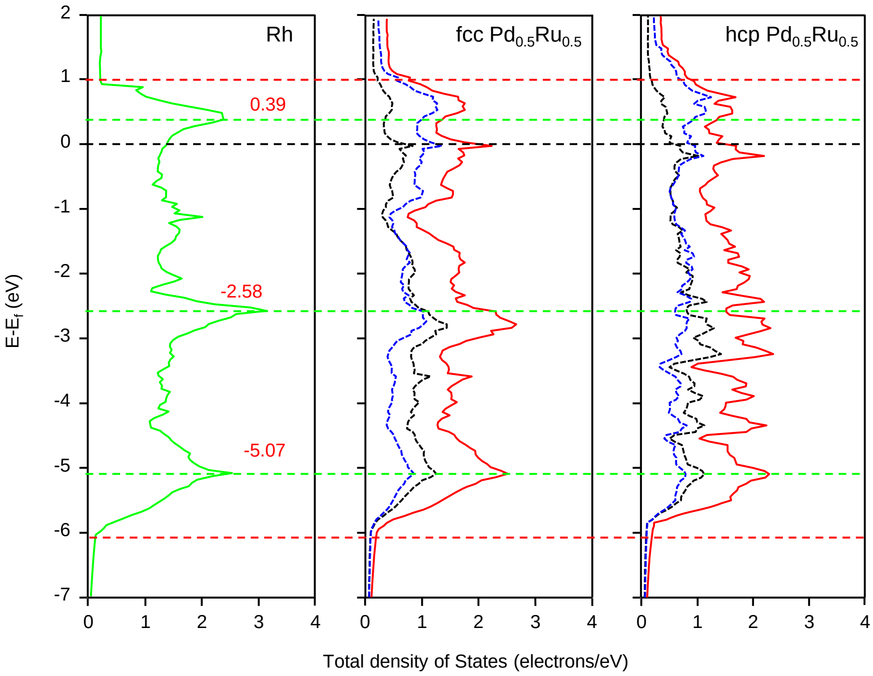

Supplementary Figure S3 | Total and partial density states of fcc and hcp$Pd_{0.5}Ru_{0.5}$. Red line, total DOS of $Pd_{0.5}Ru_{0.5}$. Black dashed line, partial DOS of Pd, Blue dashed line, partial DOS of Ru.

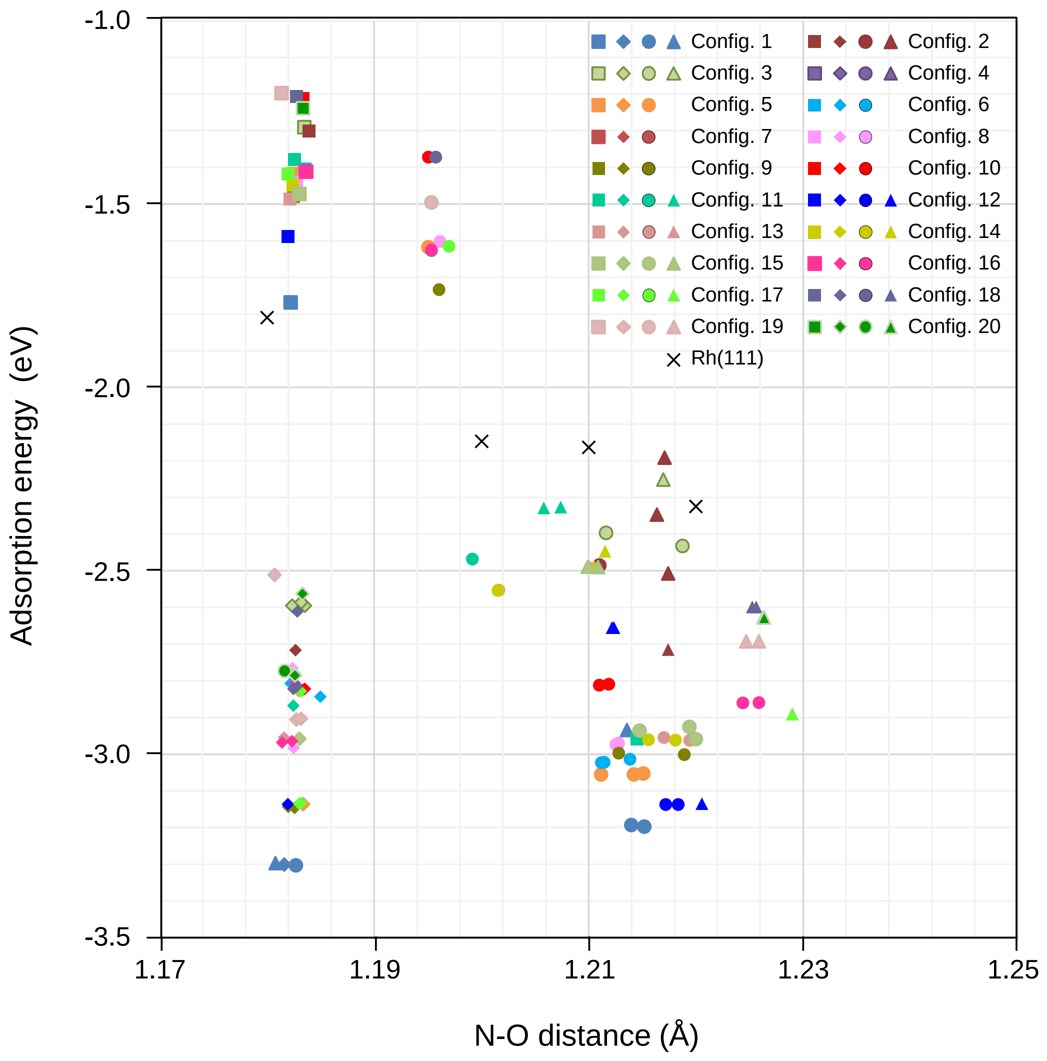

Supplementary Figure S4 | NO adsorption properties onto fcc $Pd_{0.5}Ru_{0.5}$ (111) and Rh (111). □,Pd ontop. ◇, Ru ontop. △,3fold. ○, bridge.

Influence of Pd–Ru composition on catalyst performance.

Supplementary Table S1 | Mean diameters of alloy and mono metal nanoparticles.

Composition	Mean diameter of the nanoparticle* (nm)
Ru	6.4 ± 1.7
$Pd_{0.1}Ru_{0.9}$	9.4 ± 1.7
$Pd_{0.3}Ru_{0.7}$	12.5 ± 2.2
$Pd_{0.5}Ru_{0.5}$	10.0 ± 1.2
$Pd_{0.7}Ru_{0.3}$	8.2 ± 1.6
$Pd_{0.9}Ru_{0.1}$	8.6 ± 1.4
Pd	9.8 ± 2.6

*Reproduced from Ref. [22].

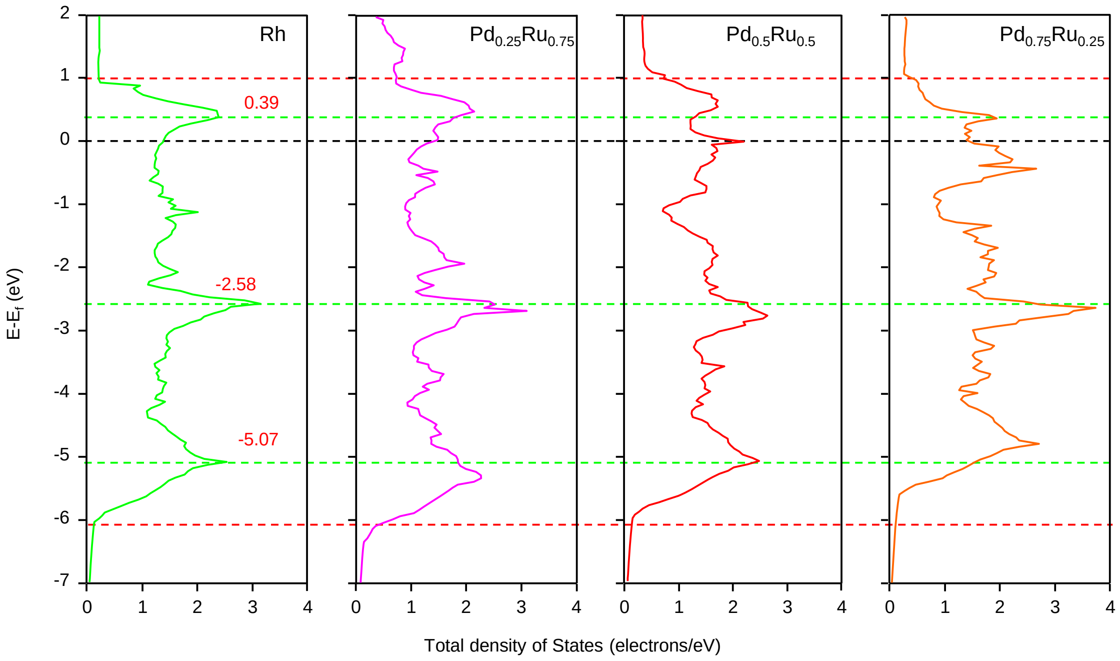

Supplementary Figure S5 | Density of states of Rh and Pd_xRu_{1-x}. x = 0.25, 0.5, 0.75.

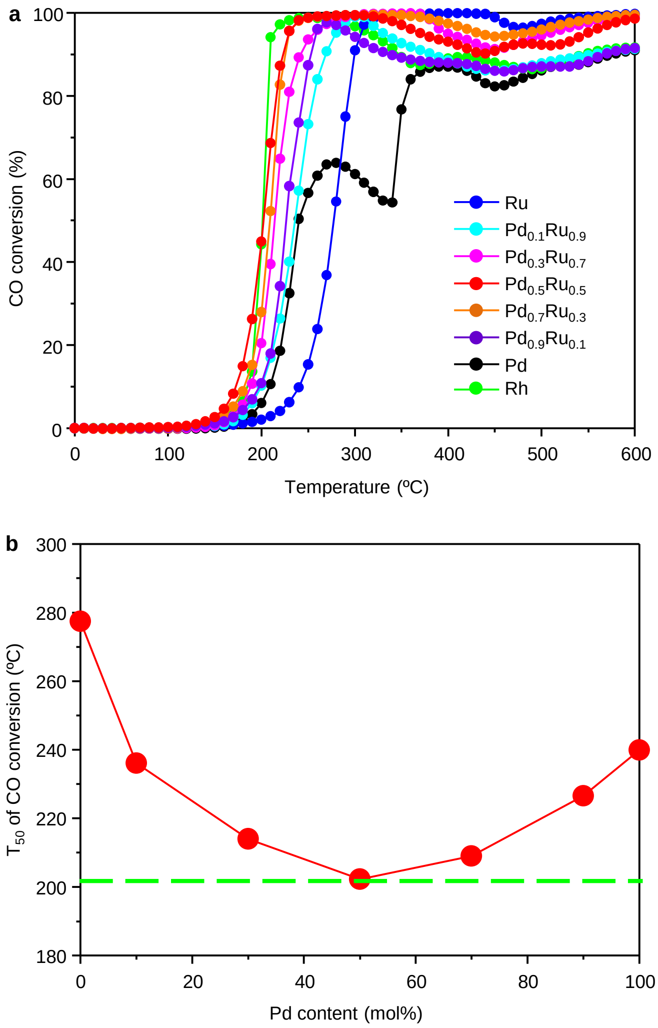

Supplementary Figure S6 | Influence of atomic ratio of Pd_xRu_{1-x} on CO purification activity. a, Temperature dependence of CO conversion for Pd_xRu_{1-x}. b, Temperatures corresponding to 50% conversion of CO_x (T_{50}) in Pd_xRu_{1-x}. The dashed line (green) is the T_{50} of Rh.

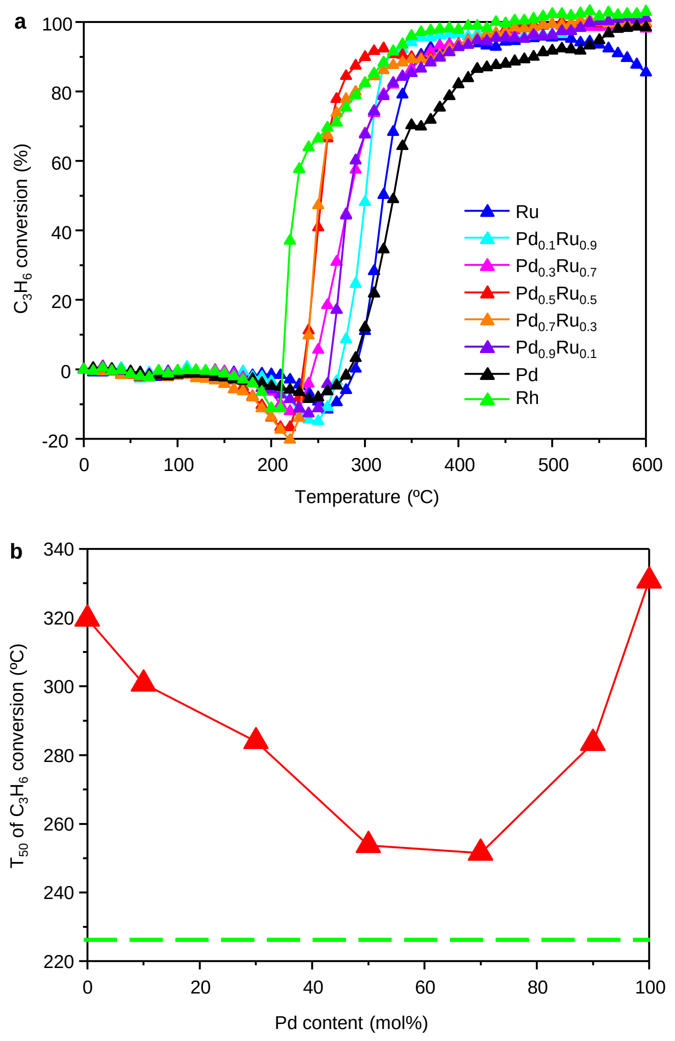

Supplementary Figure S7 | Influence of atomic ratio of Pd_xRu_{1-x} on C_3H_6 purification activity. a, Temperature dependence of C_3H_6 conversion for Pd_xRu_{1-x}. The negative conversion for each catalyst at 200 to 300 °C depends on C_3H_6 desorption from the catalyst surface. b, Temperatures corresponding to 50% conversion of C_3H_6 (T_{50}) in Pd_xRu_{1-x}. The dashed line (green) is the T_{50} of Rh.
